# Defining palliative care service capability: a scoping review to support quality improvement and benchmarking

**DOI:** 10.1007/s11136-025-04123-6

**Published:** 2026-01-09

**Authors:** Sabina Clapham, Barbara Daveson, Animut Alebel Ayalew, Kylie Draper, Anita Hartati, Kate Reed, Lisa Redwood, Leeroy William, David Currow

**Affiliations:** 1 Palliative Care Outcomes Collaboration, University of Wollongong, Mike Codd Building, Innovation Campus, Wollongong, NSW 2500 Australia; 2 Canberra Health Services, Australian Capital Territory Canberra, Australia; 3 School of Medicine, Faculty of Health, Deakin University, Melbourne, VIC Australia; 4 South West Healthcare, Warrnambool, VIC Australia; 5 College of Medicine and Public Health, Flinders University, Bedford Park, South Australia Australia

**Keywords:** Palliative care, Scoping review, Quality improvement, Service capability, Service delivery, End-of-life care

## Abstract

**Purpose:**

Palliative care is delivered across most healthcare settings, yet service capability remains poorly defined, limiting quality improvement. A clearer definition is essential to drive system-wide improvement. This scoping review was undertaken to answer two important questions: What are the key constructs that define palliative care service capability, and how can they inform quality improvement and benchmarking?

**Methods:**

A scoping review, retrieving studies from Medline, PsycINFO, and CINAHL. This scoping review was conducted in accordance with the Preferred Reporting Items for Systematic reviews and Meta-Analyses extension for scoping reviews (PRISMA-ScR). Relevant literature published in English from the year 2000 onwards, focusing on palliative care service delivery, standards, quality, and outcomes were included. Data were organised using an evidence map to define service capability and develop a conceptual framework.

**Results:**

Palliative care service capability is defined as the ability of a service to deliver care, shaped by the broader health system and organisation in which the service operates. Four core domains emerged as central to the concept and were mapped against existing standards, quality indicators and clinical frameworks: (1) assessment, planning, and care provision; (2) transitioning patients between services; (3) availability of care; and (4) collaboration and linkages across health services involved in delivering palliative care.

**Conclusion:**

This review provides a definition and conceptual model for palliative care service capability to support quality assessment and facilitate meaningful benchmarking. Integrating this framework into national quality initiatives may help identify gaps in service and system delivery, standardise care processes, and enhance patient-centred outcomes.

**Supplementary Information:**

The online version contains supplementary material available at 10.1007/s11136-025-04123-6.

## Background

The rising demand for palliative care services, driven globally by changes in the way that we die [1] and by the increasing prevalence of chronic complex illnesses, presents significant challenges for healthcare systems [2–4]. Internationally, palliative care delivery is generally categorised as specialist or generalist, with specialist palliative care services typically defined by the presence of trained staff with extensive capabilities in delivering palliative care. However, not all services can provide the same level of care and variability in service delivery contributes to the differences in care quality [3, 5, 6]. The lack of definitional clarity regarding what service capabilities are required has implications for patients and families, as inconsistent or inadequate service capability may result in variability in patient outcomes, experiences of care, and ultimately, quality of life. Even within high-income countries, marked differences in symptom outcomes have been observed between services with similar resources [7].

Given that palliative care is delivered across the continuum, from generalist to specialist services, it is essential to understand what each type of service is capable of, so that their ability to deliver care can be defined and the quality of care they provide can be compared. Previous research has described delivery features, structures, and processes [8–10], however the concept and construct of palliative care service capability remains undefined. While some countries, such as Australia, have developed capability or role delineation frameworks, these have largely focused on resource allocation rather than patient outcomes [11–15].

Palliative care standards can help describe the delivery elements or requirements of care, yet these are often both normative and aspirational and may not translate into consistent service delivery across contexts [2, 16, 17]. For instance, both the European and Australian standards emphasise access, stating that services should be available to all patients, wherever and whenever required, without delay [2, 18]. Yet not all health services are able to deliver this.

This scoping review focuses on defining and clarifying palliative care service capability as a concept and framework as it relates to the delivery of palliative care. By developing a shared construct and definition, this review aims to evaluate variations in service capability and their impacts on patient outcomes. In doing so, it aims to contribute to international quality initiatives, such as the Palliative Care Outcomes Collaboration (PCOC) and comparable programs globally, providing a framework that links service level capability with outcome measurement, improving understanding of outcome variations and enabling benchmarking of similar services. Ultimately, these efforts aim to enhance the quality of life of patients and families receiving palliative care.

## Methods

### Study design

A scoping review was used to clarify and distinguish definitions within the literature, especially when the evidence in an area is unclear [19]. Unlike a systematic review, which focuses on synthesising evidence from specific studies to answer a narrowly defined question, a scoping review allows for a broader examination of diverse sources of information. The question to be addressed was: What are the capabilities of health services for delivering palliative care?

### Data sources and search strategy

A systematic literature search was conducted in Medline, PsycINFO, and CINAHL using key terms: “*integrated care*”, “*integrated health care system*”, “*palliative care service*”, “*models of palliative care*,” “*palliative care delivery and standards*”, “*quality indicators*”, “*outcome measure and palliative care*”, “*end of life care*”, “general* palliative care”, “*primary palliative care*”, “*hospice*”. In addition, the references from the included studies were reviewed to identify further studies eligible for inclusion in the scoping review. This approach was effective given the common usage of the health workforce as a metaphor for health service delivery, such as the quantum of specialised staff or the arrangement of a palliative care team. The search period was limited to studies published from the year 2000 to 2025, to ensure relevance to current practice and contemporary models of care, as major shifts in health policy, funding and reforms have occurred since the year 2000. The search was also limited to studies published in English. Results were reported using the Preferred Reporting Items for Systematic reviews and Meta-Analyses extension for scoping reviews (PRISMA-ScR) [20].

### Eligibility and study selection

A palliative care service was defined as an organisation or clinical team delivering care with the primary purpose of optimising the quality of life for individuals with active and advanced life-limiting illnesses. Care provided by the service is delivered or informed by healthcare staff with specialised expertise in palliative care. A broad definition was used to ensure the review encompassed evidence across all palliative care settings.

Articles were included if they described elements, domains, structures, processes, or characteristics of the delivery of palliative care. However, they were excluded if they focused on single-discipline interventions, educational interventions, bereavement services (for family caregivers only), opinion articles, editorials, or conference abstracts.

The search results were downloaded and imported into EndNote bibliographic software, with duplicates removed prior to screening. Both the screening of titles and abstracts as well as the full text assessment were conducted by the primary author (SC). Where eligibility was uncertain, the study was further assessed by another reviewer (BD, AAA, KD). Eligible articles were those that included information relevant to advancing the definition of concepts and identifying constructs related to palliative care capability.

### Data extraction, summarising, and reporting the results

A structured data extraction form was developed to capture essential study information, including the first author, publication year, country where study was conducted, study aim(s), and key findings categorised across three levels: Macro (health system-level), Meso (health service-level), and Micro (clinician/discipline/patient-level). This hierarchical organisation aided understanding of how palliative care capability operates across different levels of healthcare systems.

An evidence map was constructed to visually organise the data [21]. The evidence map categorised findings into sub-groups related to service delivery, aligning them with existing frameworks for measuring palliative care delivery (Supplementary File 1). This approach facilitated the identification of constructs measured through existing frameworks, as well as gaps, patterns, and overlaps in service-level capabilities, thereby offering a structured way to assess and compare the results against established standards.

### Data synthesis

Analysis of the data involved three steps:

1. Identification and refinement of concepts, constructs and domains.

Eligible articles were systematically reviewed to identify concepts (broad ideas about capability), constructs (measurable elements), and domains (organised grouping of constructs) related to palliative care health service capability. Similar items were grouped through open coding and initial theme generation. Key constructs were further refined through iterative analysis.

2. Development of an evidence map.

An evidence map was created to visually organise the data into the broader health system concepts of capability (macro level) and the concepts of service capability (meso level). Micro-level data were excluded, as they focus on individual team members or profession-specific capabilities (e.g., medicine, social work, counselling). The evidence map also served as a tool to visually summarise relationships between service-level capabilities and their measurement (Supplementary File 1).

3. Mapping to standards, indicators, and frameworks.

Key concepts, constructs and relationships within palliative care service capability were informed by and mapped to existing quality indicators, standards and the PCOC outcome measures, as well as Australian clinical frameworks. The mapping was conducted by the research team to ensure consistency and relevance. Items were tabulated and organised into domains. The purpose of this process was to validate domains, confirm completeness, and describe their utility for operationalising capability across the continuum of palliative care service capability, ranging from generalist through to specialist care.

The primary findings were developed by SC; reviewed, clarified, and discussed by SC, BD, KR and KD; written up by SC, BD, KR, KD, AH and AAA; and further reviewed, clarified, and extended by DC.

## Results

### Search results

**Fig. 1:**
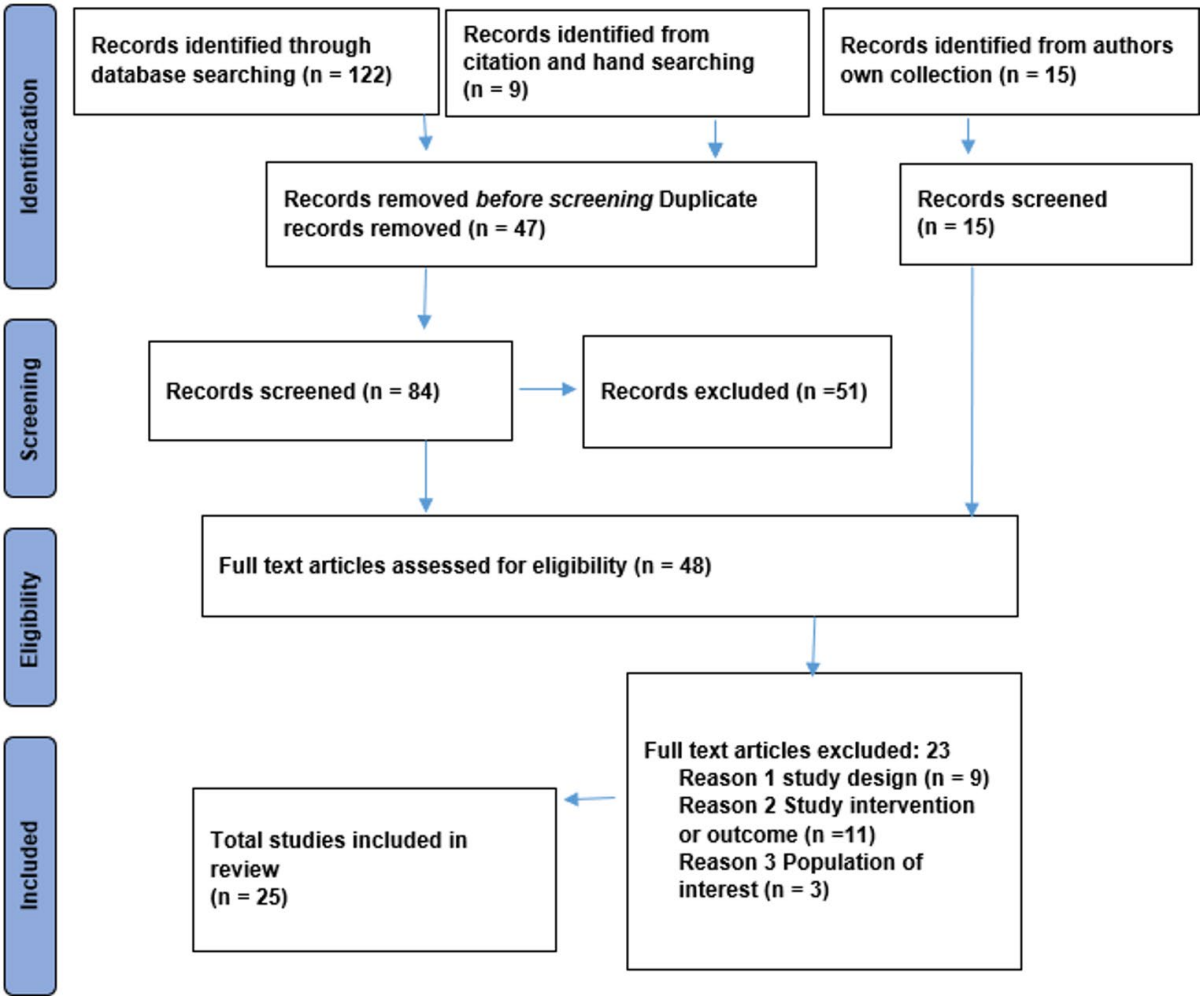
PRISMA flow diagram of study selection for a scoping review on defining concepts and identifying constructs of service capability

The initial systematic literature search yielded 146 records: 122 through database searching, nine from other sources, and 15 from the author’s own collection. After removing 47 duplicates, 99 records remained for title and abstract screening. Of these, 51 were excluded based on titles and abstracts. The remaining 48 full-text articles were assessed for eligibility. Finally, after excluding records, 25 were deemed eligible to be included (Fig. 1). The list of exluded studies can be found in Supplementary File 3.

### Characteristics of the included studies

The 25 included studies were published between 2002 and 2024. Most were multi-country (*n* = 14) [2, 22–34], followed by Australia (*n* = 4) [18, 35, 36], the United States (*n* = 3) [17, 37, 38], Canada (*n* = 2) [39, 40], England (*n* = 1) [41], and Belgium (*n* = 1) [42]. By study design, nine were reviews [22–26, 28, 33, 37, 43], four were case studies [36, 39–41], eight were consensus-based [2, 16, 17, 23, 29, 30, 32, 34], one was quantitative [35], one was based on interviews [42], one presented a conceptual framework [38], and one was a Lancet Commission report [27]. All studies addressed palliative care provision or delivery, with some focusing specifically on the integration of palliative care within health systems or health services. Data on palliative care delivery at the meso level were available from all but one study [22]. Two studies did not report data on the macro level [26, 42]. These studies were retained because they provided important factors relevant to the conceptualisation of capability and the emerging framework (Table 1).

**Table 1:** Characteristics of included studies and and their reported macro- and meso-level capabilities

Author, year, country	Type	Population	Aim	Meso level	Macro level
Arias et al. [22] 2019 Multi-country	Systematic review	54 studies included reporting on 165 indicators	To identify and conceptualise most frequently used indicators for international palliative care (PC) development reporting	–	Strategic frameworks, funding, education, access to essential medicines, and research
Bainbridge et al. [40] 2016 Canada	Case study	21 PC network administrators, 86 health care professionals, 111 carers	To evaluate the integration of PC across a network	24/7 care and volunteer support, standardised assessments, clear communication, information systems, multidisciplinary teamwork, leadership, adherence to standards, evaluation, and holistic care	Community awareness, workforce expertise, and the availability of funding
Bausewein et al. [23] 2016 Multi-country	Consensus	28 international experts across 20 European Association for Palliative Care and Board of Directors	To provide expert recommendations on outcome measurement in clinical practice and research	Outcome measurement and benchmarking, common language to describe the palliative care population and support consistent evaluation	Policymakers recommend collection of outcome data to support clinical care and research
Chen et al. [24] 2024 Multi-country	Systematic review	26 studies, older adults age ≥ 60 years approaching end of life (EoL)	To synthesise the available evidence to explore if and how Patient-Centred Outcome Measures (PCOMs) facilitate integrated PC for older people, and to build a logic model depicting the mechanisms to support integration	Integrating outcome measurement supports joint working, informs referral systems, and helps services identify gaps for development, while training and workflow adjustments are necessary to ensure effective implementation	Policy supports long-term quality evaluation of services; public awareness of PC facilitates access
Currow et al. [35] 2015 Australia	Quantitative audit	30 services, 19,747 patients	To assess whether services, patient, and caregivers’ outcomes have improved nationally since the inception of point-of care data collection, structured and timely feedback and benchmarking	Symptoms management, family carer problems, psychological and spiritual symptoms	National outcome measurement system supports high-quality palliative care
Davison et al. [36] 2002 Australia	Case study	Australian palliative care services	To examine the management of innovation by cross-functional, multi-disciplinary team (MDT) in a PC environment	MDT; information across organisations, between discipline, with carers; policies on MDT operation, exchange of information, partnering with carers, collaborating with broader health delivery system; and technology for communication and data	Understand population needs and changes in disease. Increase societal understanding of PC
Dhollander et al. [42] 2018 Belgium	Qualitative focus group interviews	6 palliative home care teams, 42 PC nurses, 7 PC physicians and 2 psychologists	To explore differences between early and late involvement and the effect on existing working procedures and skills as perceived by palliative home care teams	Early initiation of home care; frequency and planning of ongoing care; face to face and virtual assessment; psychological and family care support; advance care planning; coordination of care	–
Evans et al. [25] 2019 Multi-country (all WHO regions, predominantly USA, Europe, Western Pacific)	Rapid scoping review of systematic reviews	72 reviews, older adults age ≥ 60 years, in the last 1–2 years of life, across global health, social, and welfare services	Synthesised evidence on service delivery models (integrated geriatric and integrated PC) that optimise quality of life (QoL) at the end-of-life (EoL)	Palliative care delivered on the basis of need and benefit, not diagnosis or prognosis; comprehensive PC integrated with other specialties; use of PCOMs validated for respective population and staff engagement; MDT; integration and continuity of care across settings and specialties	Universal health coverage; national funding, accreditation and strategic frameworks; workforce development, expansion of health towards comprehensive approach in functional ability, QoL and dignified EoL
Ferrell et al. [17] 2018 USA	National consensus	American PC sector	To guide healthcare organisations and clinicians across the care continuum to integrate PC principles and best practices into their routine assessment and care of all seriously ill patients and their family caregivers	Accessible, inclusive & timely PC across all care settings; continued & coordinated care; interdisciplinary team; emotional support; continuous Quality Improvement (QI); bereavement support; availability of education and professional development	Funding for sustainability and growth; local, state and federal laws relating to advance care planning; standards of care & professional practice
Firn et al. [26] 2016 Multi-country	Narrative literature synthesis	23 studies in both hospital, community and outpatient settings	To assess the existing evidence of inpatient generalist PC providers’ perceptions of what facilitates or hinders collaboration with hospital-based specialist PC teams	Integrated model of care; skill building approach towards generalist team, professional workforce with expertise and mutual respect; proactive & timely communication, role negotiation, shared problem-solving model; MDT	–
Fulton et al. [37] 2019 USA	Systematic review & meta-analysis	Adults with advanced cancer in integrated outpatient palliative-oncology care in USA, Canada and Europe	To evaluate the effects of integrated outpatient palliative and oncology care for advanced cancer on patient and caregiver outcomes	Co-location and onsite collaboration, systems integration, holistic care by MDT, routine information exchange	Funding for workforce and co-location; push for PC beyond end-of-life care, standardises PC performance and outcome measures
Kaasa et al. [27] 2018 Multi-country	Commission report providing guidance and recommendations	Coordinated from Norway with over 30 authors across 15 countries	To transform the approach of cancer care globally by embedding PC as a core component of oncology from diagnosis through to end-of-life regardless of the patient’s prognosis or treatment intent.	Standardised care pathways that embed PC into usual oncology practice, MDT model of care, commitment from leadership and ongoing education, routine patient reported outcomes and utilise the data for improvements	Funding and infrastructure for PC services, public awareness of PC, development of global standards and international consensus on PC standards, national health systems embed PC into cancer control strategies, guidelines, funding models and accreditation standards
Kamal et al. [26] 2020 Multi-country	Scoping review	6 international experts in oncology across 5 countries	To define and advance integration frameworks by clarifying existing standards and guidelines, highlighting quality measures, identification of gaps and inconsistencies in integration and recommending improvements to quality measurement frameworks	Standardisation of care delivery, use of quality measures, team-based models of interdisciplinary care, PC provision to patient complexity	Coordinated policy action, global consensus and system wide accountability to integrate PC into oncology, organisational/institutional commitment to quality improvement
Leemans et al. [27] 2017 Multi-country	Consensus	6 European experts in PC from Belgium and The Netherlands	To develop a minimal indicator set for efficient quality assessment in PC	Physical care, coordination and continuity, family support, psychological and spiritual care, information, and care planning with both patients and their families	Nationally consistent quality indicators, policy integration into national reporting systems and frameworks, data sharing and managing gaps in measuring meaningful PC metrics, identifying systematic inequities
Luckett et al. [41] 2014 Australia	Rapid review of articles	Australian experts and opinion leaders	To evaluate existing models of PC across Organisation for Economic Co-operation and Development (OECD) countries and identify the core components that contribute to their effectiveness, to inform policy reform in Australia	Care coordination and delivery in all care settings, multidisciplinary assessment, care planning, documentation and sharing of information, deliver education and training and provide to workforce within and external to the service, participate in evaluation and research, offer bereavement services and holistic and culturally safe care	Availability and access to services via policy reform and strategic planning to enable effective models; clinical networks and funding; increase public awareness
Palliative Care Australia [16] 2018 Australia	Consensus	Australian experts and opinion leaders	To establish expectations for the range of PC services available	3 levels of PC service, symptom assessment and management 24/7, holistic and MDT, care in all settings, bereavement, advance care planning, education, collaboration, evaluation and research	Population needs, workforce availability, funding, availability across system and care setting
Payne et al. [2] 2022 multi-county (33 countries across Europe)	Three-round Delphi technique	Boards members of 52 national and professional organisations affiliated to the EAPC from 33 countries across Europe, and 13 Board Members of the EAPC.	To update the previously published standards and norms for PC in Europe, taking account of recent evidence about new practices and developments	The philosophy of PC underpins delivery; its delivery categorised by levels and delivery model; services span a range of care setting and population needs; each with specific requirements to ensure high-quality and equitable care	People have access to services; quality of service requires country level policy and strategy to ensure equity
Payne et al. [28] 2019 multi-county (Europe, USA, and Australia)	Expert consultation via face-to- face and online	33 international experts in PC and cancer care	To develop recommendations and agree on priorities for integrated PC, linked to the Integrated Palliative Care in cancer and chronic conditions	Organisational collaboration and alliance, digital information transfer across services, single point of contact at local level, clinical protocol for seamless care, needs-based referral system	Integration into national health strategies, mandatory undergraduate education, extending national PC polices beyond cancer
Ruger [36] 2010 USA	Conceptual/ theoretical framework	Not applicable	Proposed capability of health services	Health capability profile proposed as tool for services/organisations to identify gaps, includes knowledge, self-management, decision-making skills, motivation, and effective system support	Universal health insurance, public health policy, and regulation to create enabling environment
Sussman et al. [37] 2011 Canada	Comparative multiple case study	Adult cancer patients	To identify modifiable health system factors associated with population PC outcomes	Regional planning & needs assessments; demonstration projects; common chart & standardised assessments; 24/7 access; designated roles; volunteer; all care settings	Policy, strategy and funding
Sudbury-Riley et al. [39] 2021 England	Qualitative case study using service ecosystem lens	31 Patients/ families and 21 providers in Southern England	Investigated PC delivery through a service ecosystem lens	Collaboration and resource integration across hospices, general practitioners, community nurses, and care homes	Strategic plan and development of services to meet population needs
Van Riet Paap et al. [30] 2014 multi-country (12 countries)	Modified RAND Delphi consensus study	Experts in palliative cancer and dementia care (*n* = 40 panellists: 18 researchers, 22 clinicians)	To develop consensus-based international set of quality indicators (QIs) to assess the organisation of PC for cancer and dementia	Availability of PC team 24/7, access to essential palliative medicines, MDT, education/training for staff	Policy and recommendations; support for cross-national assessment
Van Riet Paap et al. [29] 2014 Multi-country (England, Germany, Italy, Norway and the Netherlands)	Semi-structured individual and focus group interviews	Professionals working in hospitals, hospices, nursing homes and primary care facilities. Interview (*n* = 40). Focus group interviews (*n* = 59).	To identify barriers to and facilitators of improvements in the organisation of PC	Culture and leadership, peer networking, mentorship and education, technology for information sharing, and availability of staff	Financial, policy, strategy and regulation at a country or regional level
Virdun et al. [31] 2018 Multi-country (15 countries)	Systematic environmental scan	National health systems in top 15 Quality of Death Index countries	To identify and describe national end-of-life care quality indicators & supporting polices.	Symptom assessment and management, holistic care including psychological, family support, information systems, MDT, bereavement services	National PC policies, standards, guidance for measurements, health financing, regulatory environment.
Woitha et al. [32] 2012 Multi-country (Europe)	Systematic review and consensus	Experts & practitioners from 7 Europe countries	Developed European set of structure/process indicators for PC	Documentation of care, equipment, continuity of care, training and appraisal of staff	Health service organisation, infrastructure, staff, education, and safety

### Capability defined

Capability was identified as the ability to deliver care that rests on the integrated resources that an organisation deliberately draws together [16, 30, 36, 39, 40], is embedded within the processes and structures of the health systems and depends on the interactions of individuals working within these systems and services [26, 36, 41]. The findings further extend the concept by highlighting capability of health systems and organisations to describe, plan, and commission services [11, 44].

### A conceptual model of palliative care capability

**Fig. 2:**
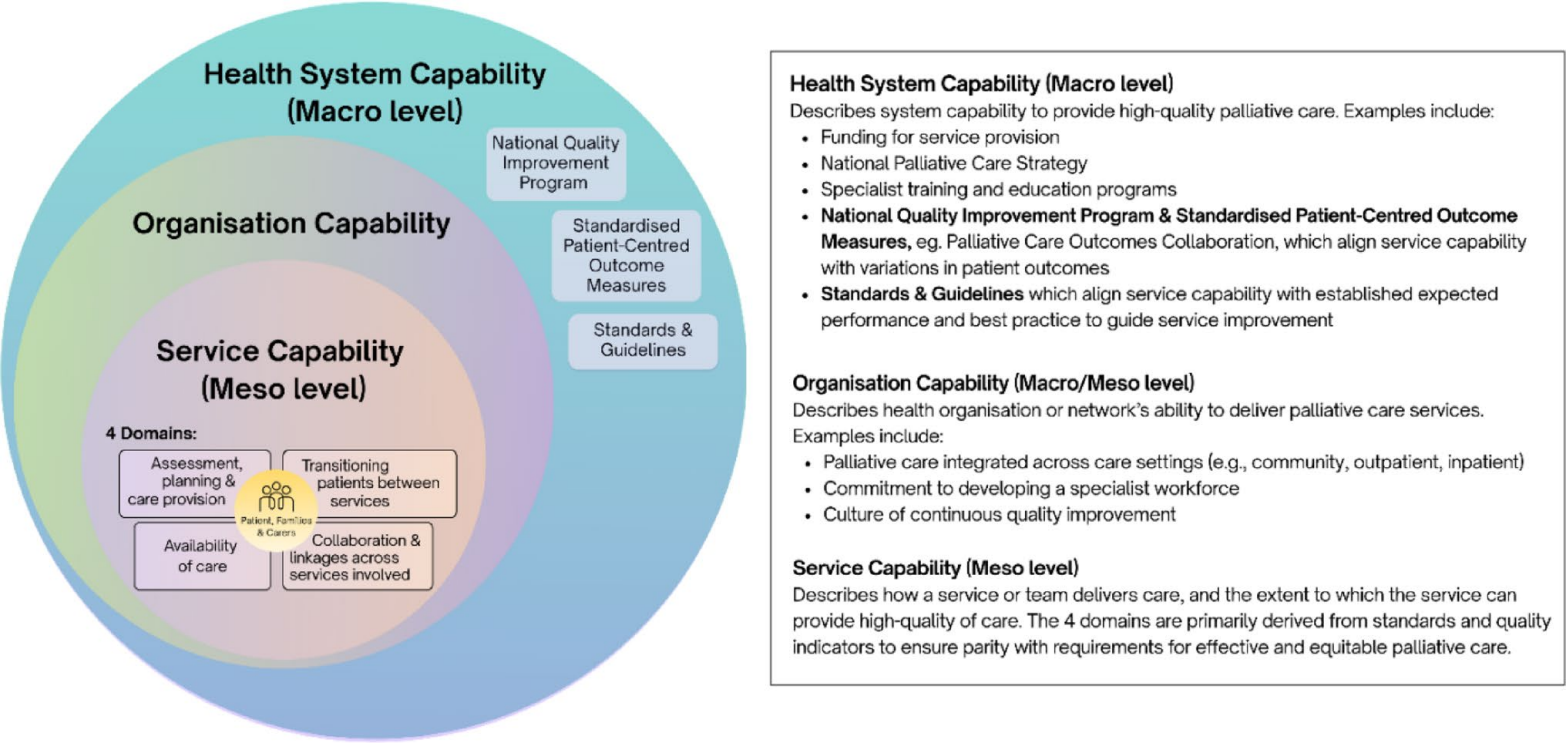
Conceptual model of service capability, showing its alignment with quality indicators and standards, and its interrelationships with health system and organisational level capabilities

Palliative care capability describes the role, ability, and resources of a health service to deliver palliative care. At the macro level, health system capabilities encompassed palliative care policy, strategy, and funding [22, 25, 30, 37, 43]. At the organisational or network level, it included the purposeful integration and availability of palliative care across all care settings, alongside an organisational commitment to develop and retain a specialist workforce and engage in quality improvement [18, 24, 28, 30, 34]. At the meso-level, four key domains of palliative care capability emerged, each addressing distinct aspects of service delivery: (1) assessment, planning, and care provision; (2) availability and accessibility of care; (3) transitioning patients between services; and (4) collaboration and linkages across services. The interrelationships across levels are presented in Fig. 2, which illustrates the conceptual model of service capability and its alignment with quality indicators and standards, showing how health system, organisational, and service-level capabilities interact.

The four domains of service capability are described in Table 2. They were derived primarily from standards and quality indicators, ensuring the model reflects established quality indicators and standards required for effective and equitable palliative care delivery, as shown in Table 3. The detailed mapping of these standards and indicators is available as a Supplementary File 2.

**Table 2:** Four domains of palliative care service capability, and their definitions

Domain	Definition
Assessment, planning, and care provision	This domain encompasses the systematic assessment and reassessment of patient and family needs, that underpins the planning and provision of patient- and family-centred palliative care [17, 18, 25, 40]. It includes the use of validated assessment tools and outcome measures to systematically identify and monitor care needs to enhance the quality and responsiveness of care [23–25, 35], utilising family meetings as integral part of planning and establishing goals of care [18, 25, 27, 42], provision of grief and bereavement care [2, 17, 18, 32, 33, 43], as well as applying clinical protocols, guidelines, and to promote continuous improvement and integration of palliative care into patient’s healthcare [17, 28, 30, 40]. Delivering this care requires a multidisciplinary approach and engaging trained volunteers [22, 39, 40]
Availability of care	This domain refers to the availability and accessibility of palliative care across all care settings that is responsive to needs [2, 17, 18, 25, 30, 43]. It includes after-hours care (24/7), access to diverse care models (such as consultative or outpatient services) [18, 32, 39, 40, 43], and access to essential palliative care medicines [22, 31]
Transitioning patients in and between services based on assessed need	This domain covers the coordination and continuity of care as patients move within and between services. It is supported by standardised and documented processes (including referral criteria, admission and discharge protocols), specialist liaison to address patient needs, and communication systems that ensures care plans are consistently shared across providers [2, 17, 24, 26, 27, 29, 30, 36, 37, 40, 42, 43]
Collaboration and linkages (between services and clinicians)	This domain encompasses the collaboration and linkages between services and clinicians to facilitate the integration of palliative care [25, 28, 30, 37, 41, 43]. It involves mentorship and partnership with clinicians and services working alongside specialist palliative care to build capacity to deliver palliative care, as well as fostering interdisciplinary research and knowledge development [2, 23–26, 40, 41]

### Measuring capability to support quality improvement and benchmarking

The mapping confirmed that the four service capability domains are well-aligned with existing palliative care standards, quality indicators, and clinical frameworks, providing a sound foundation for developing a measurement framework (Table 2). Because capability reflects the actual ability of a service to deliver care, measurement focuses on what services provide in practice rather than what they aspire to or are expected to deliver (Table 3). A measurement framework may enable services to be categorised along a continuum from generalist to specialist palliative care, providing more nuanced grouping for benchmark comparisons. For example, at the generalist level, a health service may deliver selected elements of Domain 1, such as discussing goals of care, documenting a palliative care plan, and recognising the dying phase. In contrast, the most comprehensive specialist palliative care is able to deliver all elements across the four domains as described in Table 2.

The mapping also revealed that, although most Australian clinical frameworks specify minimum requirements for the safe delivery of clinical services [11, 12], they differ in intent and conceptual underpinning. Some frameworks incorporate aspirational targets, supporting service development and enhancement beyond minimum standards [13].

Importantly, aligning the capability framework with PCOC quality indicators provides a direct connection between service capability and patient-centred outcomes. This link was most evident in Domain 1 (Assesment Planning and Provision), Domain 2 (Availability of Care), and Domain 3 (Collaboration and Linkages), demonstrating how the ability of a service is related to patient outcomes (Supplementary File 4).

**Table 3:** Definition of capability and its distinction from standards, quality indicators, and clinical frameworks

Concept	Definition	Construct of health service measured
Standards for palliative care	Standards provide frameworks for operationalising care and promoting uniformity in service delivery across settings [17, 18, 28]. They are used to measure performance within, and between care settings [2, 17]. Standards are generally normative but may incorporate aspirational components to support those services with growing capacity. Palliative care standards provide guidance for best practice palliative care to ensure high quality care is delivered. Assessment against palliative care standards is usually voluntary by self-assessment. [2, 17, 28]	Performance
Quality indicators for palliative care services	Quality indicators measure healthcare processes, structures, and outcomes in different healthcare systems and countries [22, 28, 29, 34]. Indicator sets inform patients, providers, and policymakers on the quality of care. They identify areas for quality improvement and can be used for benchmarking [33, 35]. Use of quality indicators is usually voluntary [33]. Indicators are usually assessed through multiple data sources (e.g., patient, family, and staff) and include numerators and denominators and may be setting or speciality specific [23, 32, 35]	Care quality
Clinical role delineation and clinical capability frameworks for palliative care services	In Australia, these frameworks have guided the clinical and capital service planning since the mid-1980s, primarily focusing on risk management and resource stratification. All Australian jurisdictions delineate levels of care, resources, and staffing capabilities [12–15]. Assessment of a health services’ clinical role is through self-assessment [13]. They are most frequently used at a district or organisational level. Countries other than Australia have adopted clinical role or capability frameworks (sometimes referred to as role delineation or service-levels) [44]. These frameworks aim to provide a consistent language and descriptors that healthcare providers and planners use in the strategic development of palliative care [16]	Patient safety
New finding - palliative care service capability	Service capability refers to the role, ability, and resources of a health service to deliver palliative care across key domains, including assessment, care planning, provision of care, patient transitions, availability of care, and collaboration between services. It encompasses the processes, structures, and workforce necessary to deliver care effectively. Workforce is an integral component of service capability, as it directly impacts the quality of care	Ability to deliver care

## Discussion

This scoping review synthesised evidence from 25 studies to clarify and distinguish the capabilities required by health services to deliver palliative care. It identified key concepts, constructs and domains that define capability, resulting in a new definition of palliative care service capability. Drawing on these findings, the review proposes a conceptual model of palliative care service capability that integrates three interrelated levels of the health system.

At the health system level, capability is shaped by enabling policies, strategic direction, and sustainable funding mechanisms that support equitable access to palliative care [17, 22, 25, 43]. At the organisational or network level, capability encompasses the deliberate integration of palliative care across settings, the availability of a skilled and supported workforce, and a culture of continuous quality improvement [23, 28, 35, 40]. At the service level, capability was defined through four domains that capture essential elements of service delivery: (1) assessment, planning, and care provision; (2) availability and accessibility of care; (3) transitions and continuity of care; and (4) collaboration and linkages across services.

The conceptual model provides a foundation for assessing service capability, guiding quality improvement, and grouping comparable services for meaningful benchmarking. It offers an explanatory basis for variations in patient outcomes among services with similar resources. For example, access to 24/7 palliative care is associated with improved symptom outcomes and quality of life [45, 46], yet its provision remains highly variable and inequitable [47]. Identifying which services provide 24/7 care not only helps explain outcome variation but also strengthens advocacy to governments and funders to address service gaps. This is particularly valuable when embedded within national quality initiatives such as PCOC.

The capability framework addresses a critical gap in existing instruments, as current standards, quality indicators, and clinical role delineation frameworks each provide only partial insights into service delivery. Australian clinical frameworks come closest to capturing aspects of capability, but they primarily define minimum requirements for patient safety rather than the full spectrum of high-quality palliative care, and they vary in purpose [11–13, 15]. Evidence from the PCOC initiative demonstrates that the use of patient-centred outcome measures - a core feature of the capability framework particularly across the domains of assessment, planning, care provision and collaboration across services - is linked to improved patient outcomes [48]. This suggests that improving the capabilities of palliative care services is feasible and may support better patient outcomes and quality of care.

### Strengths and limitations

A key strength of this study is its novelty in defining and clarifying palliative care service capability, a concept with limited presence in the literature. A scoping review was therefore selected to explore and map the concept of palliative care capability as it applies to health systems and services. The scoping review design enabled the inclusion of diverse sources, allowing concepts, constructs, and domains to be examined across system, organisational, and service levels. The use of an evidence map and alignment with existing standards, quality indicators, and clinical frameworks strengthened the transparency and applicability of the findings, providing a practical foundation for quality improvement and benchmarking initiatives.

Several limitations should also be noted. Most of the evidence included in this review primarily reflects the perspectives of clinicians, which may not capture all aspects of service capability. Consequently, important dimensions of high-quality care from the viewpoint of those receiving it may be underrepresented. Incorporating the views of patients and families in future research is essential to ensure that the framework reflects the elements of service delivery most meaningful to them, such as adequate equipment and safety [49] which align with the capability domains of *Assessment*,* Planning and Provision of Care* and *Availability of Care*. For instance, the service has the capability to assess occupational, functional and physical needs and is able to provide the equipment necessary for functional performance. Including these perspectives in future work would enhance the validity, relevance, and comprehensiveness of the capability framework.

Methodological constraints also remain. The concept of “health service capability” had not been previously defined, and the search strategy may have missed relevant studies. In addition, study selection bias is possible, as the study selection process was conducted by primary author (SC) with consultation only in uncertain cases. These methodological constraints should be considered when interpreting the findings.

Finally, health system capability is influenced by external factors such as national policies, funding structures, and workforce development strategies, which directly influence a service’s ability to deliver care. While these influences are acknowledged within the capability model, the framework does not currently provide a mechanism for their assessment.

## Conclusions

This scoping review has defined the concept and constructs of palliative care service capability, offering a framework to better understand health services’ ability to deliver high-quality palliative care. By distinguishing service capability from quality indicators, standards, and clinical frameworks our findings provide a pathway for evaluating how capability influences patient outcomes. The conceptual model developed can be integrated into national quality initiatives such as the PCOC to support ongoing improvements in service delivery, care quality, and benchmarking. To strengthen the concept of service capability and its measurement, future research should focus on incorporating essential features described by patients, families, and carers. Additionally, further studies are required to assess the feasibility, reliability, and validity of measuring palliative care service capability to ensure its practical application across diverse healthcare settings.

## Supplementary Information

Below is the link to the electronic supplementary material.

Supplementary Material 1
