# Supplementary material for: Defining palliative care service capability: a scoping review to support quality improvement and benchmarking
Source: Qual Life Res. 2026 Jan 9;35(2):38. doi: 10.1007/s11136-025-04123-6 (PMC12789151; doi:10.1007/s11136-025-04123-6)
Supplement: Supplementary file 1 — Supplementary Material 1 [file 11136_2025_4123_MOESM1_ESM.pdf]

Supplementary file

Mapping stage 1: capability domains derived from established Quality Indicator domains and Standards of care

**Capability domains**	**Quality Indicators (Q-PAC /Belgium) [1, 2]**	**Quality indicators (IMPACT/Europal) [3, 4]**	**Quality indicators (Measuring What Matters USA) [5]**	**European standards for delivering palliative care (EAPC) [6]**	**Palliative care standards Clinical Practice Guidelines USA [7]**	**Palliative care standards Australia [8]**
Assessment, planning, and care provision	Physical aspects of care	Documentation of care	Structures and process of care	Across four domains	Physical Aspects of Care	Assessment of needs
	Coordination and continuity of care	Equipment and continuity of care	Physical aspects of care	Delivery- MDT and volunteers	Psychological and Psychiatric Aspects	Developing the care plan
	Care for the family		Psychological and Psychiatric Aspects	Across all four domains	Social Aspects of Care	Caring for carers
	Psychological and spiritual aspects of care		Spiritual, Religious, and Existential Aspects of Care	PC services, philosophy	Spiritual, Religious, and Existential Aspects of Care	Providing care
	Information and care planning with patient			PC service delivery and PC services	Care of the Patient Nearing the End of Life	Grief support
	Information and care planning with family			Palliative care services		
Transition in and between teams	N/A	N/A	N/A	Levels- interaction between teams, networks, specialties and levels	N/A	Transitions within and between teams
Capacity building	N/A	N/A	N/A	Philosophy, levels of PC, PC service delivery	Structure and Processes of Care	Quality improvement
Availability of care	N/A	N/A	N/A	Delivery and PC services	Structure and Processes of Care	N/A
Domains not mapped to capability to deliver palliative care (process, structure, workforce)	Communication and information needs with other care givers (1 item mapped)	Training and appraisal of staff	Ethical and Legal Aspects of Care	Terminology and definitions	Ethical and Legal Aspects of Care Cultural Aspects of Care	Staff qualifications and training
	Circumstances surrounding death	Availability of controlled drugs				Service culture (the concept underpinning this domain is infrastructure)

Mapping stage 2: capability items mapped to existing quality improvement programs in Australia (Standards and Outcomes)

Domain	Palliative Care Capability Framework for delivering quality care	PCA National Palliative Care Standards [9]	PCOC Outcome measures and quality improvement indicators [10]	PCOC Implementation framework for outcome measurement [11]
Assessment, planning and provision of care	Goals of care are discussed, and a palliative care plan is agreed upon and documented	**Standard 2:** The person, their family and carers work in partnership with the PC team to communicate, plan, set goals of care and support informed decisions about the care plan. ***2.4., 2.8.***	N/A	N/A
	Family meetings or case conferences are provided in accordance with documented care plans	**Standard 3**: The person's family and carers needs are assessed and directly inform provision of appropriate support and guidance about their role. ***3.1., 3.2., 3.3., 3.4., 3.7.***	N/A	N/A
	Bereavement information is provided for family/carers	**Standard 6**: Families and carers have access to bereavement support services and are provided with information about loss and grief. ***6.1., 6.4., 6.7., 6.8.***	N/A	N/A
	A specialist grief and bereavement service, which includes routine and ongoing assessment of bereavement needs			
	The terminal (dying) phase of care is identified, and care is provided to meet the needs of the patient and their family/carer	**Standard 1**: Initial and ongoing assessment incorporates the person's physical, psychological, cultural, social and spiritual experiences and needs.<br>*1.3.*	Number of deaths outside terminal phase.	**Key Strategy** 2: processes in place to support routine and multidisciplinary outcome measurement assessment ***2.1, 2.2, 2.3, 2.4, 2.5, 2.6***<br>**Key strategy 3**: Outcome measurement and the response framework is included in orientation and ongoing training for medical, nursing and allied health staff ***3.1***
	Palliative care assessment tools and outcome measures are used at every patient and family/carer interaction to measure and evaluate the management of symptoms and problems	**Standard 1**: Initial and ongoing assessment incorporates the person's physical, psychological, cultural, social and spiritual experiences and needs. **1.1.** ***1.2. 1.3. 1.4.1.5. 1.6.***<br>**Standard 2**: The person, their family and carers work in partnership with the PC team to communicate, plan, set goals of care and support informed decisions about the care plan. ***2.1. 2.9.***<br>**Standard 4**: The provision of care is based on the assessed needs of the person, informed by evidence, and is consistent with the values, goals and preferences of the person as documented in their care plan. ***4.5.***	Number of benchmarks met.<br><br>Number of patients with moderate to severe symptoms and number of family/carers with moderate to severe problem.<br><br>Trends in symptom and problem outcomes	
	Identified psychosocial and spiritual care needs are addressed by a suitably qualified staff member other than a nurse or medical officer (with no specialist training in bereavement care)			
	Interdisciplinary assessment, care and care coordination by the specialist palliative care team including medical, nursing and allied health staff			
	Care planning and delivery involves extensive access to allied health services to meet a broad range of physical, functional, psychosocial, spiritual and bereavement needs			
	Patients and family/carers are supported by palliative care trained volunteers	**Standard 9:** Staff and volunteers are appropriately qualified, are engaged in continuing professional development and are supported in their roles ***9.4., 9.7.***	N/A	N/A
	Care is supported by clinical protocols, guidelines, and policy specific to palliative and end-of-life care facilitating consistency in clinical practice and integration of palliative care	**Standard 7**: The service has a philosophy, values, culture, structure and environment that supports the delivery of person-centred palliative care ***7.2.***	Participation in PCOC benchmarking.<br><br>Continuous or sustained improvement in patient outcomes.	***Key Strategy 1:*** leadership support for outcome measurement (including PROMS) and the embedment of an assessment and response protocol is secured, both at an organisational and service level (leadership staff include the appropriate medical, nursing and allied health leads, and quality managers) ***1.1 1.4***
	Engagement in quality improvement by participation in measuring and evaluating patient and family/carer outcomes	**Standard 4**: The provision of care is based on the assessed needs of the person, informed by evidence, and is consistent with the values, goals and preferences of the person as documented in their care plan. ***4.6.***	Participation in PCOC benchmarking.<br><br>Continuous or sustained improvement in patient outcomes.	**Key Strategy 5:** *Engagement in quality improvement by participation in measuring and evaluating patient and family/carer outcomes*

		**Standard 8**: Services are engaged in quality improvement and research to improve service provision and development ***8.1., 8.2., 8.4., 8.6.***		***5.1, 5.2, 5.3, 5.4,***
Responsiveness	After-hours advice and support is provided (non-specialist or specialist health care professional or team)	**Standard 2**: The person, their family and carers work in partnership with the PC team to communicate, plan, set goals of care and support informed decisions about the care plan. ***2.10.***,<br>***Standard 4****: The provision of care is based on the assessed needs of the person, informed by evidence, and is consistent with the values, goals and preferences of the person as documented in their care plan.* ***4.1., 4.4., 4.5.***<br>3.5.	Number of responsive care benchmarks met.<br>Number of patients with moderate to severe symptoms.	**Key Strategy 2**: processes in place to support routine and multidisciplinary outcome measurement assessment ***2.1***<br>**Key Strategy 5:** *Engagement in quality improvement by participation in measuring and evaluating patient and family/carer outcomes*
	Specialist palliative care support and advice is provided through telephone or video conferencing 24 hours, and 7 days a week			
	Specialist palliative care medicine and nursing support and advice is provided in person, 24 hours, and 7 days per week			
	In reach consultation specialist palliative care service is provided for one-off or ongoing assessment (inside local health services area or organisation)	**Standard 5**: Care is integrated across the person's experience to ensure seamless transitions within and between services **5.7.**	Number of service settings of care participating in PCOC.<br><br>Number patients waiting longer than 48hrs.	N/A
	Outreach consultation specialist palliative care service is provided for one-off or ongoing assessment (outside local health service area or organisation)			N/A
Collaboration and linkages	A communication system is in place to share care planning and assessment information within and between services	**Standard 2**: The person, their family and carers work in partnership with the PC team to communicate, plan, set goals of care and support informed decisions about the care plan ***2.5., 2.7.***<br>***Standard 5****: Care is integrated across the person's experience to ensure seamless transitions within and between services* ***5.2.***	Number of service settings of care participating in PCOC	**Key Strategy 2**: processes in place to support routine and multidisciplinary outcome measurement assessment ***2.3, 2.4***<br>***Key Strategy 1:*** leadership support for outcome measurement (including PROMS) and the embedment of an assessment and response protocol ***1.4***
	Documented protocols to ensure patients are transitioned into and out of the service. This involves referral, triage and discharge. It includes transitions involving emergency departments, other specialities and primary care	***Standard 5:*** *Care is integrated across the person's experience to ensure seamless transitions within and between services* ***5.3., 5.4., 5.6., 5.8****.*	Outcome measure: timely commencement of palliative care<br><br>Number of patients waiting longer than 48hrs	Key Strategy 5: *Engagement in quality improvement by participation in measuring and evaluating patient and family/carer outcomes*<br>*EF 5.4-*
	Formal links to other services providing palliative care to ensure patients with changing needs, goals of care, and requiring transitions between settings of healthcare receive specialist palliative care medical and nursing advice and support	**Standard 5**: Care is integrated across the person's experience to ensure seamless transitions within and between services ***5.1., 5.2., 5.7.***	N/A	N/A
Capacity building and quality improvement	Provide palliative care advice, education and mentorship to clinicians and other services	**Standard 8:** Services are engaged in quality improvement and research to improve service provision and development **8.*8.,***<br>**Standard 9:** Staff and volunteers are appropriately qualified, are engaged in continuing professional development and are supported in their roles ***9.4.***	N/A	N/A
	Provide clinical placements for health care professionals as part of their professional development or ongoing formal studies in in palliative care	**Standard 8**: Services are engaged in quality improvement and research to improve service provision and development **8.8**<br>**Standard 9:** Staff and volunteers are appropriately qualified, are engaged in continuing professional development and are supported in their roles ***9.3., 9.4***	N/A	N/A
	Provide specialist palliative care interdisciplinary training to those working within the service and for those working in other services	**Standard 9**: Staff and volunteers are appropriately qualified, are engaged in continuing professional development and are supported in their roles ***9.5, 9.6.***	N/A	**Key strategy 3:** Outcome measurement and the response framework is included in orientation and ongoing training for medical, nursing and allied health staff ***3.1***

	Lead, design, implement and participate in palliative care specific research and publish findings with demonstrable impact	**Standard 8:** Services are engaged in quality improvement and research to improve service provision and development<br>***8.7.***	N/A	**Key Strategy 5**: *Engagement in quality improvement by participation in measuring and evaluating patient and family/carer outcomes* ***5.4***

Mapping stage 3: capability items and levels of palliative care service mapped to QLD, NSW, VIC, PCA

Mapping palliative care capability domains, items and service levels					
**Capability Domain**	***Capability items***	**QLD capability framework [12]**	**NSW role delineation framework [13]**	**Victorian capability framework [14]**	**PCA level [15]**
Assessment, planning, and care provision	Goals of care are discussed and a palliative care plan is agreed upon and documented	1	-	1	-
	The terminal (dying) phase of care is identified and care is provided to meet the needs of the dying patient and their family/carer	-	-	-	-
	Family meetings or case conference are provided in accordance with documented care plans	1	-	1	-
	Bereavement information and support is provided for all families	3	-	1	-
	Palliative care assessment tools and outcome measures are used at every patient/carer interaction to measure and evaluate the management of symptoms and problems	-	-	2	-
	Identified psychosocial, spiritual and bereavement care needs are responded to by a staff member with specialist training in bereavement care or someone who is equipped to provide bereavement support	3	3	2	2
	Care planning and delivery involves extensive access to allied health services in order to meet a broad range of physical, functional, psychosocial, spiritual and bereavement needs	1	3	2	3
	Care is supported by clinical protocols, guidelines and policy specific to palliative and end-of-life care facilitating consistency in clinical practice and integration of palliative care	-	-	2	-
	Interdisciplinary assessment and care planning by the specialist palliative care team including medical, nursing and allied health staff	4	4	3	3
	Patients and carers are supported by palliative care trained volunteers	-	-	3	-
	A specialist grief and bereavement service, which includes routine and ongoing assessment of bereavement needs	5	-	3	-
	Engage in continuous improvement to improve palliative care patient outcomes	-	-	2	-
Transition in and between teams	A communication system in place to share care planning and assessment information within and between services	6 (network level)	-	2	-
	Documented protocols and pathways to ensure patients are transitioned into and out of the service. This involves referral, triage and discharge. It includes transitions involving emergency departments, other specialities and primary care	6 (network level)	-	3	-
	Formal links to other services providing palliative care to ensure patients with changing needs, goals of care, and requiring transitions between settings of healthcare receive specialist palliative care medical and nursing advice and support.	6	5	3	3
Availability of care	After-hours advice and support is provided (non-specialist or specialist health care professional or team)	3	3	1	-
	Specialist palliative care support and advice is provided through telephone or video conferencing 24 hours, 7 days a week	5	5	2	3
	In reach consultation specialist palliative care service is provided for one-off or ongoing assessment (inside local health services area or organisation)	4	4	3	-
	Outreach consultation specialist palliative care service is provided for one-off or ongoing assessment (outside local health service area or organisation).	4	4	3	-

	Specialist palliative care medicine and nursing support and advice is provided in person, 24 hours, and 7 days per week	6	6	3	3
Collaboration and linkages between services and clinicians)	Provides palliative care support, education and mentorship to clinicians and services (1, 2, 3 capabilities)	-	-	3	3 (2)
	Provides clinical placements for health care professionals as part of their professional development or formal studies in palliative care	-	-	2	-
	Builds awareness of and advocates for palliative care in the wider community	-	-	3	3
	Provide specialist palliative care interdisciplinary training to those working within the service and for those working in other services	-	-	2	3
	Lead, design, implement and participate in palliative care specific research and publish findings	-	-	2	3

References

1. Leemans, K., et al., *Systematic Quality Monitoring For Specialized Palliative Care Services: Development of a Minimal Set of Quality Indicators for Palliative Care Study (QPAC).* Am J Hosp Palliat Care, 2017. **34**(6): p. 532-546.
2. Cohen, J., et al., *Nationwide evaluation of palliative care (Q-PAC study) provided by specialized palliative care teams using quality indicators: Large variations in quality of care.* Palliative Medicine, 2021: p. 02692163211019881.
3. Woitha, K., et al., *Validation of quality indicators for the organization of palliative care: a modified RAND Delphi study in seven European countries (the Europall project).* Palliative medicine, 2014. **28**(2): p. 121-129.
4. Woitha, K., et al., *Development of a set of process and structure indicators for palliative care: the Europall project.* BMC health services research, 2012. **12**: p. 381.
5. Dy, S.M., et al., *Measuring what matters: top-ranked quality indicators for hospice and palliative care from the American Academy of Hospice and Palliative Medicine and Hospice and Palliative Nurses Association.* J Pain Symptom Manage, 2015. **49**(4): p. 773-81.
6. Payne, S., et al., *Revised recommendations on standards and norms for palliative care in Europe from the European Association for Palliative Care (EAPC): A Delphi study.* **0**(0): p. 02692163221074547.
7. Ferrell, B.R., et al., *National Consensus Project Clinical Practice Guidelines for Quality Palliative Care Guidelines, 4th Edition.* J Palliat Med, 2018. **21**(12): p. 1684-1689.
8. Australia, P.C., *National palliative care standards.* Canberra: Palliative Care Australia, 2018.
9. Palliative Care Australia, *National palliative care standards*, in *Canberra: Palliative Care Australia*. 2018.
10. Kobel C, B.S., Redwood L, Clapham S, Daveson B *Patient Outcomes in Palliative Care – Australian National report, July – December 2023. Palliative Care Outcomes Collaboration*. 2024, Faculty of Science, Medicine and Health, University of Wollongong: Wollongong Australia.
11. Clapham, S.M.F., Kable, Le-Tisha; Joseph Natalie; Connolly Jane, *Implementing the Palliative Care Outcomes Collaboration (PCOC): a guide for services 2nd edition*. 2020, University of Wollongong: Wollonong
12. Health, Q. *Clinical Services Capability Framework*. 2018 1 June 2021]; Available from: https://www.health.qld.gov.au/clinical-practice/guidelines-procedures/service-delivery/cscf.
13. Health, N.M.o. *NSW Health Guide to the Role Delineation of Clinical Services*. 2019.
14. Victoria, G.H. *Palliative Care Service Capability Framework*. 2016.
15. Palliative Care Australia, *Palliative care service development guidelines.* Palliative Care Australia, 2018.

Supplementary file: Capability items and levels mapped to Australian capability and role delineation frameworks

In Australia, these frameworks have guided the clinical and capital service planning since the mid-1980s, with the primary focus on risk management and resource stratification. All Australian jurisdictions delineate levels of care (service levels), resources, and staffing capabilities [1-3]. Countries other than Australia have also adopted clinical role or capability frameworks (sometimes referred to as role delineation or service levels). These frameworks aim to provide a consistent language and descriptors that healthcare providers and planners use in the strategic development of palliative care [1, 4-7].

Table 1: Description of capability frameworks across Australian states and territories

Jurisdiction	Document or framework name	Description	Palliative care capability levels in health services* -
Australian Capital Territory	ACT Health Services Plan	Defines the minimum requirements for support services, workforce, and infrastructure necessary to deliver safe and effective care. The framework provides a common language for describing and coordinating health services, supports decision-making in service planning, and categorises service complexity from No Planned Service (NPS) to Level 6 (complex service capability), linked to the availability of clinical support services such as pathology, pharmacy, anaesthetics, imaging, allied health, and operating theatres.	6
New South Wales	Role Delineation of Clinical Services	Provides a framework outlining the minimum support services, workforce, and other requirements for the safe delivery of clinical services. It delineates service levels and offers a consistent language for describing clinical services across NSW. The Guide is used by Local Health Districts (LHDs) and Specialty Health Networks (SHNs) for service planning and development and assists clinical governance in assessing risks and determining the services provided by health facilities.	6
Queensland	Clinical Services Capability Framework	A framework for Public and Licensed Private Health Facilities sets the minimum standards for support services, staffing, safety, and other requirements to ensure safe clinical services in Queensland. It establishes capability requirements for acute and sub-acute services and provides a consistent language for service planning and development. The framework includes modules for each service area, specifying service levels for both public and private facilities. The framework describes clinical services by capability level and applies to the planning and provision of services in public and private hospitals or day hospitals.	6

South Australia	Clinical Services Capability Framework – Palliative Care	A guide for coordinating and integrating health service planning and delivery across South Australia. It supports public health facilities by outlining clinical capability criteria, service requirements, workforce needs, and interdependencies between clinical areas to ensure safe, high-quality care. The framework ensures Local Health Networks develop consistent, localised clinical service plans while complementing policies and models of care.	6
Tasmania	Tasmanian Role Delineation Framework and Clinical Services Profile	A statewide clinical service planning tool for Tasmania's public health system. It provides a transparent, evidence-based framework to guide the planning, development, and coordination of safe and sustainable health services. The framework ensures that each level of clinical service provision is supported by corresponding clinical support services, staff profiles, and minimum standards to deliver care safely and efficiently. By categorising service levels and defining requirements, the framework safeguards patient safety, facilitates clinical risk management, and supports effective statewide coordination and consistent models of care.	4
Victoria	Palliative Care Service Capability Framework	Defines a health service's safe scope of practice based on its physical and human resources, including workforce skills, infrastructure, equipment, clinical support services, and governance. It establishes minimum requirements for delivering safe and effective care, ensuring services operate within their capabilities and refer patients to higher-level care when necessary. The framework provides a consistent approach to planning, service development, and risk management at local, regional, and system levels, supporting transparency and safety across the healthcare system.	3
Western Australia	WA Health Clinical Services Framework	Guides planning, budgeting, and developing health services and adaptable models of care across Western Australia. It defines service capabilities, workforce and infrastructure needs, and technology integration to meet evolving healthcare demands, ensuring safe, high-quality, and accessible care while optimising resources and aligning with WA Health's strategic goals.	6

*Australian frameworks classify service levels based on clinical, workforce, and service delivery capabilities

Table 2: Levels of palliative care service capability mapped: QLD, NSW, VIC, Palliative Care Australia (national).

PCOC capability framework			Mapping service levels*			
**Capability Domain**	***Capability items***	**Potential level of PC service**	**QLD framework**	**NSW framework**	**Victorian framework**	**Palliative Care Aust guideline**
Assessment, planning, and care provision	Goals of care are discussed and a palliative care plan is agreed upon and documented	1	1	-	1	-
	The terminal (dying) phase of care is identified and care is provided to meet the needs of the dying patient and their family/carer	1	-	-	-	-
	Family meetings or case conference are provided in accordance with documented care plans	2	1	-	1	-
	Bereavement information and support is provided for all families	2	3	-	1	-
	Palliative care assessment tools and outcome measures are used at every patient/carer interaction to measure and evaluate the management of symptoms and problems	3	-	-	2	-
	Identified psychosocial, spiritual and bereavement care needs are responded to by a staff member with specialist training in bereavement care or someone who is equipped to provide bereavement support	3	3	3	2	2
	Care planning and delivery involves extensive access to allied health services in order to meet a broad range of physical, functional, psychosocial, spiritual and bereavement needs	3	1	3	2	3
	Care is supported by clinical protocols, guidelines and policy specific to palliative and end-of-life care facilitating consistency in clinical practice and integration of palliative care	3	-	-	2	-
	Interdisciplinary assessment and care planning by the specialist palliative care team including medical, nursing and allied health staff	4	4	4	3	3
	Patients and carers are supported by palliative care trained volunteers	4	-	-	3	-
	A specialist grief and bereavement service, which includes routine and ongoing assessment of bereavement needs	4	5	-	3	-
	Engage in continuous improvement to improve palliative care patient outcomes	4	-	-	2	-
Transition in and between teams	A communication system in place to share care planning and assessment information within and between services	4	6 (network level)	-	2	-
	Documented protocols and pathways to ensure patients are transitioned into and out of the service. This involves referral, triage and discharge. It includes transitions involving emergency departments, other specialities and primary care	5	6 (network level)	-	3	-
	Formal links to other services providing palliative care to ensure patients with changing needs, goals of care, and requiring transitions between settings of healthcare receive specialist palliative care medical and nursing advice and support.	6	6	5	3	3
Availability of care	After-hours advice and support is provided (non-specialist or specialist health care professional or team)	2	3	3	1	-
	Specialist palliative care support and advice is provided through telephone or video conferencing 24 hours, 7 days a week	4	5	5	2	3
	In reach consultation specialist palliative care service is provided for one-off or ongoing assessment (inside local health services area or organisation)	4	4	4	3	-
	Outreach consultation specialist palliative care service is provided for one-off or ongoing assessment (outside local health service area or organisation).	6	4	4	3	-

	Specialist palliative care medicine and nursing support and advice is provided in person, 24 hours, and 7 days per week	6	6	6	3	3
Collaboration and linkages between services and clinicians)	Provides palliative care support, education and mentorship to clinicians and services (1, 2, 3 capabilities)	4	-	-	3	3 (2)
	Provides clinical placements for health care professionals as part of their professional development or formal studies in palliative care	4	-	-	2	-
	Builds awareness of and advocates for palliative care in the wider community	4	-	-	3	3
	Provide specialist palliative care interdisciplinary training to those working within the service and for those working in other services	6	-	-	2	3
	Lead, design, implement and participate in palliative care specific research and publish findings	6	-	-	2	3

***** The service levels defined in the frameworks are mapped to compare similarities and differences, with the potential to align a service level within the conceptual capability framework.

1. Health, Q. *Clinical Services Capability Framework*. 2018 1 June 2021]; Available from: https://www.health.qld.gov.au/clinical-practice/guidelines-procedures/service-delivery/cscf.
2. Health, N.M.o. *NSW Health Guide to the Role Delineation of Clinical Services*. 2019.
3. Department of Health & Human Services, V., *Victoria's end of life and palliative care framework.* 2016.
4. Australia, P.C., *Palliative Care Service Development Guidelines*. 2018: Canberra, Australia.
5. Health, M.o. *Resource and Capability Framework for Integrated Adult Palliative Care Services in New Zealand.* . 2012 [cited March 2021]; Available from: https://www.health.govt.nz/system/files/documents/publications/resource-capability-framework-adult-palliative-care-services-v2.pdf.
6. Ryan, K. *Role Delineation Framework for Adult Palliative Care Services*. 2015 [cited March 2021]; Available from: https://www.lenus.ie/handle/10147/622898.
7. Duckett, S., *Hospital role delineation: the technology and the politics.* The International journal of health planning and management, 1991. **6**(2): p. 133-142.

Supplementary File 4: Capability items and levels mapped to Australian capability and role delineation frameworks

In Australia, these frameworks have guided the clinical and capital service planning since the mid-1980s, with the primary focus on risk management and resource stratification. All Australian jurisdictions delineate levels of care (service levels), resources, and staffing capabilities [1-3]. Countries other than Australia have also adopted clinical role or capability frameworks (sometimes referred to as role delineation or service levels). These frameworks aim to provide a consistent language and descriptors that healthcare providers and planners use in the strategic development of palliative care [1, 4-7].

Table 1: Description of capability frameworks across Australian states and territories

Jurisdiction	Document or framework name	Description	Palliative care capability levels in health services* -
Australian Capital Territory	ACT Health Services Plan	Defines the minimum requirements for support services, workforce, and infrastructure necessary to deliver safe and effective care. The framework provides a common language for describing and coordinating health services, supports decision-making in service planning, and categorises service complexity from No Planned Service (NPS) to Level 6 (complex service capability), linked to the availability of clinical support services such as pathology, pharmacy, anaesthetics, imaging, allied health, and operating theatres.	6
New South Wales	Role Delineation of Clinical Services	Provides a framework outlining the minimum support services, workforce, and other requirements for the safe delivery of clinical services. It delineates service levels and offers a consistent language for describing clinical services across NSW. The Guide is used by Local Health Districts (LHDs) and Specialty Health Networks (SHNs) for service planning and development and assists clinical governance in assessing risks and determining the services provided by health facilities.	6
Queensland	Clinical Services Capability Framework	A framework for Public and Licensed Private Health Facilities sets the minimum standards for support services, staffing, safety, and other requirements to ensure safe clinical services in Queensland. It establishes capability requirements for acute and sub-acute services and provides a consistent language for service planning and development. The framework includes modules for each service area, specifying service levels for both public and private facilities. The framework describes clinical services by capability level and applies to the planning and provision of services in public and private hospitals or day hospitals.	6

South Australia	Clinical Services Capability Framework – Palliative Care	A guide for coordinating and integrating health service planning and delivery across South Australia. It supports public health facilities by outlining clinical capability criteria, service requirements, workforce needs, and interdependencies between clinical areas to ensure safe, high-quality care. The framework ensures Local Health Networks develop consistent, localised clinical service plans while complementing policies and models of care.	6
Tasmania	Tasmanian Role Delineation Framework and Clinical Services Profile	A statewide clinical service planning tool for Tasmania's public health system. It provides a transparent, evidence-based framework to guide the planning, development, and coordination of safe and sustainable health services. The framework ensures that each level of clinical service provision is supported by corresponding clinical support services, staff profiles, and minimum standards to deliver care safely and efficiently. By categorising service levels and defining requirements, the framework safeguards patient safety, facilitates clinical risk management, and supports effective statewide coordination and consistent models of care.	4
Victoria	Palliative Care Service Capability Framework	Defines a health service's safe scope of practice based on its physical and human resources, including workforce skills, infrastructure, equipment, clinical support services, and governance. It establishes minimum requirements for delivering safe and effective care, ensuring services operate within their capabilities and refer patients to higher-level care when necessary. The framework provides a consistent approach to planning, service development, and risk management at local, regional, and system levels, supporting transparency and safety across the healthcare system.	3
Western Australia	WA Health Clinical Services Framework	Guides planning, budgeting, and developing health services and adaptable models of care across Western Australia. It defines service capabilities, workforce and infrastructure needs, and technology integration to meet evolving healthcare demands, ensuring safe, high-quality, and accessible care while optimising resources and aligning with WA Health's strategic goals.	6

*Australian frameworks classify service levels based on clinical, workforce, and service delivery capabilities

1. Health, Q. *Clinical Services Capability Framework*. 2018 1 June 2021]; Available from: https://www.health.qld.gov.au/clinical-practice/guidelines-procedures/service-delivery/cscf.
2. Health, N.M.o. *NSW Health Guide to the Role Delineation of Clinical Services*. 2019.
3. Department of Health & Human Services, V., *Victoria's end of life and palliative care framework.* 2016.
4. Australia, P.C., *Palliative Care Service Development Guidelines*. 2018: Canberra, Australia.
5. Health, M.o. *Resource and Capability Framework for Integrated Adult Palliative Care Services in New Zealand.* . 2012 [cited March 2021]; Available from: https://www.health.govt.nz/system/files/documents/publications/resource-capability-framework-adult-palliative-care-services-v2.pdf.
6. Ryan, K. *Role Delineation Framework for Adult Palliative Care Services*. 2015 [cited March 2021]; Available from: https://www.lenus.ie/handle/10147/622898.
7. Duckett, S., *Hospital role delineation: the technology and the politics.* The International journal of health planning and management, 1991. **6**(2): p. 133-142.

Supplementary file 3. List of excluded studies

Study	Reason (design, intervention or outcome, population)
Wilson NJ, Kizer KW. Oncology management by the "new" Veterans Health Administration. Cancer. 1998;82(10 Suppl):2003-9.	io
Brenne A-T, Knudsen AK, Raj SX, Skjelvan L, Lund J-Å, Thronæs M, et al. Fully Integrated Oncology and Palliative Care Services at a Local Hospital in Mid-Norway: Development and Operation of an Innovative Care Delivery Model. Pain and therapy. 2020;9(1):297-318.	io
Evans JM, Mackinnon M, Pereira J, Earle CC, Gagnon B, Arthurs E, et al. Building capacity for palliative care delivery in primary care settings: Mixed-methods evaluation of the INTEGRATE Project. Canadian family physician Medecin de famille canadien. 2021;67(4):270-8.	io
Fainsinger RL, Romer AL. Addressing Quality of Life at the Edmonton Palliative Care Program. Journal of Palliative Medicine. 2001;4(3):417-22.	io
Ferrell BR, Chung V, Koczywas M, Smith TJ. Dissemination and Implementation of Palliative Care in Oncology. Journal of clinical oncology : official journal of the American Society of Clinical Oncology. 2020;38(9):995-1001.	s
Hewison A, Hodges E, Balasubramanian S, Swani T. System transformation in palliative and end of life care: developing a model for excellence. Journal of health organization and management. 2021;ahead-of-print(ahead-of-print).	s
Jones L, Candy B, Davis S, Elliott M, Gola A, Harrington J, et al. Development of a model for integrated care at the end of life in advanced dementia: A whole systems UK-wide approach. Palliative medicine. 2016;30(3):279-95.	s
Mittmann N, Liu N, MacKinnon M, Seung SJ, Look Hong NJ, Earle CC, et al. Does early palliative identification improve the use of palliative care services? PloS one. 2020;15(1):e0226597.	io
Robinson L, Poole M, McLellan E, Lee R, Amador S, Bhattarai N, et al. Supporting good quality, community-based end-of-life care for people living with dementia: the SEED research programme including feasibility RCT. 2020.	io
Seow H, Snyder CF, Mularski RA, Shugarman LR, Kutner JS, Lorenz KA, et al. A framework for assessing quality indicators for cancer care at the end of life. Journal of Pain and Symptom Management. 2009;38(6):903-12.	io
Vaughan L, Bardsley M, Bell D, Davies M, Goddard A, Imison C, et al. Models of generalist and specialist care in smaller hospitals in England: a mixed-methods study. 2021.	io
Morrison RS. A national palliative care strategy for Canada. Journal of palliative medicine. 2018 Jan 1;21(S1):S-63.	s
Loucka M, Payne S, Brearley S, IMPACT E. How to measure the international development of palliative care? A critique and discussion of current approaches. Journal of pain and symptom management. 2014 Jan 1;47(1):154-65.	s
Tanuseputro P, Budhwani S, Bai YQ, Wodchis WP. Palliative care delivery across health sectors: a population-level observational study. Palliative medicine. 2017 Mar;31(3):247-57.	io
Collins A, Brown JE, Mills J, Philip J. The impact of public health palliative care interventions on health system outcomes: a systematic review. Palliative Medicine. 2021 Mar;35(3):473-85.	p

Lawrence, E., Massey, A., Whatley, V., Nicholls, L., Hay, F., & Willis, D. (2024). Increasing the number of adults on a palliative care end-of-life register: a quality improvement project. British Journal of Healthcare Management, 30(8), 1–9. https://doi.org/10.12968/bjhc.2023.0088	s
Ahluwalia SC, Harris BJ, Lewis VA, Colla CH. End-of-life care planning in accountable care organizations: associations with organizational characteristics and capabilities. Health Services Research. 2018 Jun;53(3):1662-81.	s
Hepgul N, Wilson R, Yi D, Evans C, Bajwah S, Crosby V, et al. Immediate versus delayed short-term integrated palliative care for advanced long-term neurological conditions: the OPTCARE Neuro RCT. 2020.	p
Dudgeon D. The Impact of Measuring Patient-Reported Outcome Measures on Quality of and Access to Palliative Care. J Palliat Med. 2018;21(S1):S76-S80.	io
Kinchin I, Kelley S, Meshcheriakova E, Viney R, Mann J, Thompson F, et al. Cost-effectiveness of a community-based integrated care model compared with usual care for older adults with complex needs: a stepped-wedge cluster-randomised trial. Integrated healthcare journal. 2022;4(1):e000137.	s
Kinley J, Preston N, Froggatt K. Facilitation of an end-of-life care programme into practice within UK nursing care homes: A mixed-methods study. International Journal of Nursing Studies. 2018;82:1-10.	s
Lanoix M. The ethics of imperfect cures: models of service delivery and patient vulnerability. Journal of medical ethics. 2013;39(11):690-4.	p
Lovell MR, Luckett T, Boyle FM, Phillips J, Agar M, Davidson PM. Patient education, coaching, and self-management for cancer pain. Journal of clinical oncology : official journal of the American Society of Clinical Oncology. 2014;32(16):1712-20.	io

Supplementary file 1: map of the evidence related to palliative care service capability

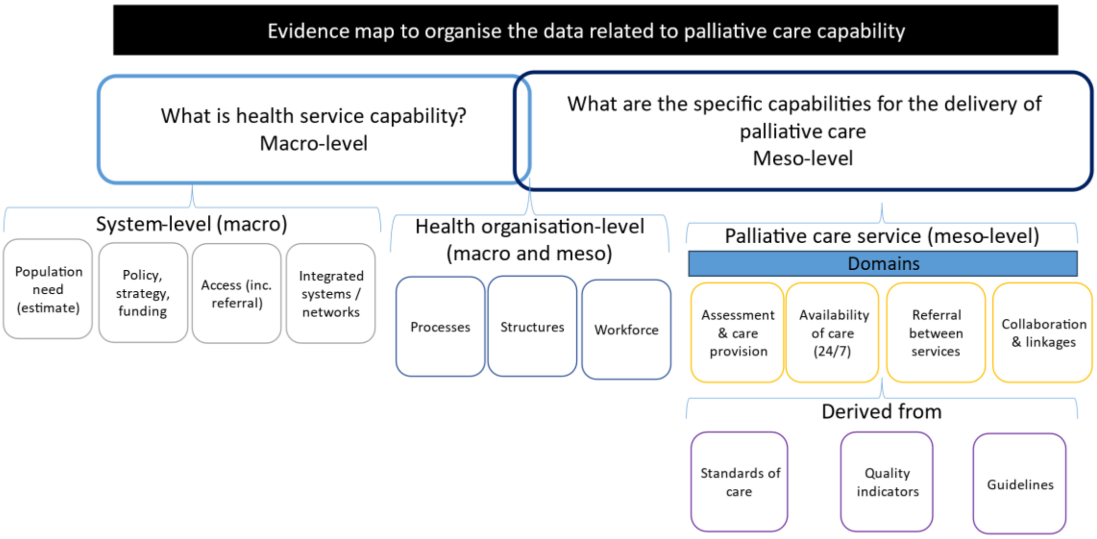

Meso (e.g. what the capability framework intends to capture)
Definition: Organisational or service-delivery structures that bridge system-level policy with frontline care.

Application: Focus on hospitals, hospices, primary care networks and community organisations—how they're structured, resourced and governed to interpret and implement policy. For example, two hospitals with comparable budgets might deliver symptom assessments differently because of variations in leadership style, workflows or IT infrastructure.

Macro
Definition: Broad, system-wide forces such as national health policy, funding models, legislation, accreditation, and cultural norms.

Application: examine how national strategies, reimbursement structures, or international standards shape the overall availability and prioritisation of services. For example, whether palliative care is included in universal health coverage, or how workforce shortages are influenced by national training pipelines.

Micro
Definition: The direct experiences of patients, families, and clinicians at the point of care.

Application: Explore how care is delivered and received, from clinician–patient communication and culturally appropriate practices to shared decision-making and tool use in consultations. For example, whether a needs-assessment tool is routinely adopted and how patients perceive its value.

Palliative care service capability
Service capability refers to the role, ability, and resources of a health service to deliver palliative care across key domains, including assessment, care planning, provision, patient transitions, availability of care, and collaboration between services. It encompasses the processes, structures, and workforce necessary to deliver care effectively. Workforce is an integral component of service capability, as it directly impacts the quality of care
